# Tobacco mosaic virus infection triggers an RNAi-based response in *Phytophthora infestans*

**DOI:** 10.1038/s41598-019-39162-w

**Published:** 2019-02-25

**Authors:** Tiziana Mascia, Rossella Labarile, Fiona Doohan, Donato Gallitelli

**Affiliations:** 1 Dipartimento di Scienze del Suolo della Pianta e degli Alimenti, Università degli Studi di Bari Aldo Moro, Bari, Italy; 2 Istituto del CNR per la Protezione Sostenibile delle Piante, UOS di Bari, Bari, Italy; 3 School of Biology & Environmental Science, University College, Dublin, Belfield Dublin 4, Ireland

## Abstract

RNA interference (RNAi) is a sequence identity-dependent RNA degradation mechanism conserved in eukaryotic organisms. One of the roles of RNAi is as a defense system against viral infections, which has been demonstrated in filamentous fungi but not in oomycetes. We investigated the virus-RNAi interplay in the oomycete *Phytophthora infestans* using a crucifer-infecting strain of the plant virus tobacco mosaic virus (TMVcr) and its derivative TMVcr-Δ122 that is mutated in the sequence of the p122 replicase subunit and thus inhibited in RNA suppression activity. In this study we provide evidence that replication of TMVcr-Δ122 but not of TMVcr was impaired in *P*. *infestans* as well as in tobacco plants used as positive control. The interference was associated with induction of high transcription of dicer-like genes *Pidcl*2 and *NtDCL*2 and of RNA-dependent-RNA-polymerase *Pirdr*1 and *NtRDR*1 in *P*. *infestans* and tobacco, respectively. These high transcription levels suggest an RNAi-based response that TMVcr-Δ122 mutant was not able to suppress. Taken altogether, results of this study demonstrated that an antiviral silencing activity operates also in *P*. *infestans* and that a plant virus could be a simple and feasible tool for functional studies also in oomycetes.

## Introduction

RNA interference (RNAi) is a sequence identity-dependent RNA degradation mechanism involved in the innate immunity response in plants, invertebrates, fungi and oomycetes. Double-stranded RNAs (dsRNA) derived from transgenes, endogenous genes, viruses and transposable elements activate the mechanism by processing the dsRNAs into 21–30 nucleotides (nt) long duplexes, called primary small interfering RNAs (siRNA) via a type III ribonuclease of the Dicer-like (DCL) protein family. Then members of the Argonaute protein (AGO) family recruit siRNAs to assemble RNA-induced silencing complexes (RISCs) that are the effectors of the process. RISCs remove one of the two strands from primary siRNAs and use the other as a sequence-specific guide to direct degradation or silencing of complementary RNAs in different subcellular compartments^1–4^. In some organisms, a host RNA-dependent RNA polymerase (RdRp) uses the single-stranded RNA (ssRNA) templates removed from the primary siRNAs to synthesize mobile secondary siRNA duplexes for amplification and spread of the silencing signal throughout host tissues^5,6^.

Filamentous fungi and oomycetes host the replication of a large number of mycoviruses and oomycete viruses, the majority of which have dsRNA genomes although an increasing number of viruses with plus-sense ssRNA genomes^7–10^ and sequence similarities with plant viruses is being reported^7^. Thus, a population of virus-derived small interfering RNAs (vsiRNAs) to target viral nucleic acid would be a predictable component also of fungi and oomycetes RNAi pathways in response to the accumulation of genomic dsRNA or of dsRNA intermediates of viral replication^11–16^. The importance of RNAi as defense mechanism against viruses is reflected in the fact that most viruses encode RNAi silencing suppressors (VSRs) that interfere with different but specific steps of the silencing pathway^17^ or contribute to keep a balance between host development and virus accumulation levels^18^. The use of counter defense strategies based on suppression of RNAi has been documented for the S10 gene of the Rosellinia necatrix mycoreovirus 3 (RnMyRV3) and for the p29 multifunctional protein encoded by Cyphonectria hypovirus 1(CHV1-EP713)^19,20^ but to the best of our knowledge to date this evidence has not been provided for oomycetes. The *Rosellinia necatrix/*RnMyRV3 and *Cyphonectria parasitica/*CHV1 systems are robust models for studying host defense-viral counterdefense interactions in filamentous fungi. RnMyRV3 is an encapsidated dsRNA mycoreovirus replicating inside the virions and its nucleic acid is never exposed to cytoplasm while CHV1-EP713 is a naked ssRNA hypovirus. Despite this remarkable difference both the viruses are sensitive to RNAi and express a VSRs^16^. The suppressor activity of the S10 gene coded by RnMyRV3 was demonstrated in a *R*. *necatrix* strain carrying a constitutively silenced green florescent protein (GFP). In this host, RnMyRV3 infection reduced the accumulation of siRNAs derived from GFP and increased the accumulation of GFP dsRNA, suggesting an interference with the dicing step of *R*. *necatrix* RNAi^21^. Agroinfiltration in plants validated the VSR role of the S10 gene of RnMyRV3^21^. Similarly, the papain-like protease P29 encoded by the hypovirus CHV1-EP713 suppressed RNAi either in *C*. *parasitica* or in plant. The P29 product is phylogenetically and functionally affine to potyvirus HC-Pro as a protease, a symptom determinant, and a VSR^12^.

Gene silencing in oomycetes is most well characterized in the potato late blight pathogen *Phytophthora infestans* that encodes the three core components involved in RNAi comprising two DCL genes (*Pidcl*1, *Pidcl*2), five AGO genes (*Piago*1–5) and one RNA-dependent RNA polymerase gene (*Pirdr*1)^22,23^. Comparative genomics led to identification in *P*. *infestans* of other genes potentially involved in gene silencing such as class 1 RNase III, chromodomain proteins, DEAD-box helicases, histone deacetylases and histone methyltransferases, coming to the conclusion that proteins similar to Dicer, Ago and Histone deacetylases are essential for gene silencing in the oomycete^23^.

This study investigated the RNAi-based response of the oomycetes *P*. *infestans* to the infection of the plant virus tobacco mosaic virus and its mutant defective in the ability of the p122 protein (TMVcr-Δ122) to suppress RNAi^24^. In particular, in this manuscript we show that TMVcr and its VSRs-defective mutant are able to replicate and express in *P*. *infestans* and that, similarly to plants, the p122 VSRs coded by TMVcr functions also in this oomycete to suppress RNAi triggered by viral infection. The rationale for using a plant virus derives from the observation that TMV could enter and persist in the mycelia of the soil-inhabiting phytopathogenic oomycete *Pythium* sp.^25^ and particles of TMV and tobacco necrosis virus could be transfected in *P*. *arrehnomanes*^26^. These early studies were followed up and more thoroughly discussed in a recent paper^27^ where it was demonstrated that a wild-type isolate of TMV was able to enter, replicate and persist up to seventh subculture in cells of the phytopathogenic fungi *Colletotrichum acutatum*, *C*. *clavatum* and *C*. *theobromicola* and that *C. acutatum* responded to the TMV infection by activating an RNAi-based mechanism. Compared to current strategies to investigate RNAi in filamentous fungi and oomycetes, the approach used in this study is more direct, easy to do, and feasible since it simply entailed the addition of a purified virus preparation to liquid cultures of *P*. *infestans*.

## Results and Discussion

### TMV enters and expresses in mycelia of *P*. *infestans*

**Figure 1:**
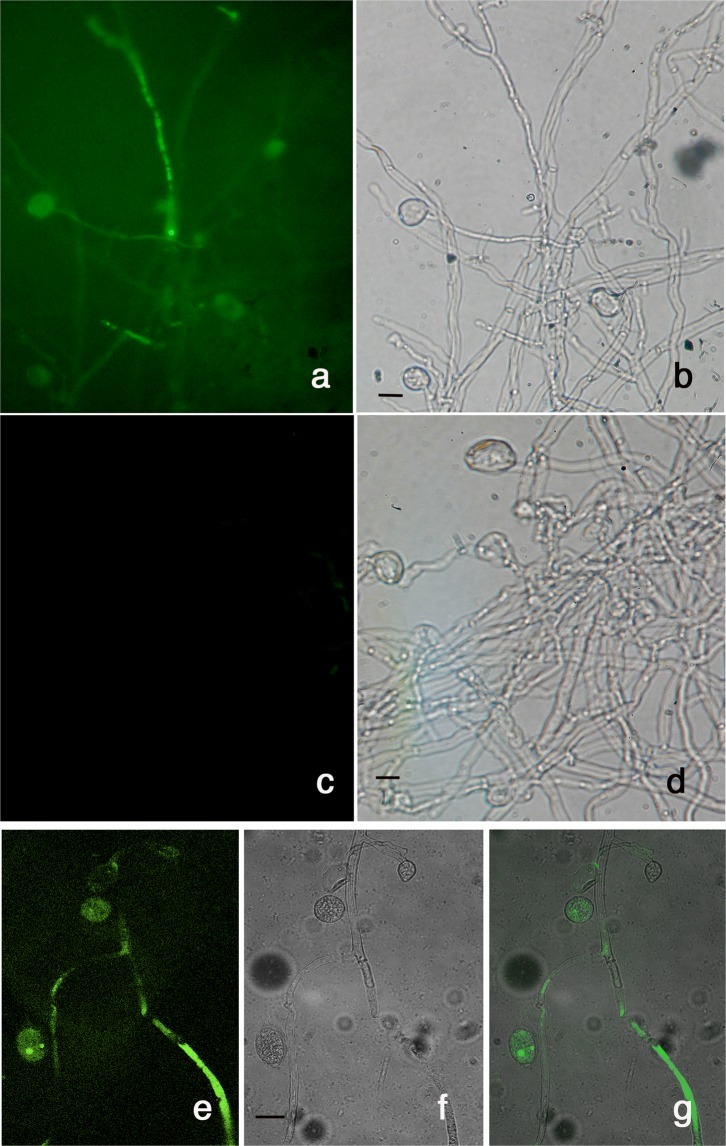
The tobacco mosaic virus-based recombinant vector TMV-GFP-1056 expresses in *P*. *infestan*s. Epifluorescence and confocal microscopy of *P*. *infestans* at 10 dpi with TMV-GFP-1056 viewed under UV (**a**) and white light (**d**). (**c** and **d**) Images of WT *P*. *infestans* not exposed to viral co-incubation. Confocal microscopy of the same sample viewed under UV (**e**) and white light (**f**) and merged (**g**). Scale bars 20 μm.

In a preliminary study, we assessed the ability of TMV to enter, replicate and express in mycelia of *P*. *infestans*. Oomycete liquid cultures were inoculated with a purified preparation of the TMV-based vector TMV-GFP-1056, expressing green fluorescent protein (GFP) as reporter. Before mycelia inoculation, the vector was tested in tobacco plants to verify the biological expression of GFP (see Supplementary Fig. S1) after which, it was used for the inoculation of liquid cultures of *P*. *infestans*. Observation under an epifluorescence microscope showed strong and diffuse green fluorescence in hyphae and sporangia collected at 10 dpi with TMV-GFP-1056 (Fig. 1a) but not in mycelia not exposed to viral inoculum (Fig. 1c). Further observation with a confocal microscope confirmed that fluorescence localized in hyphae and sporangia and very likely expressed also in zoospores of *P*. *infestans* inoculated with TMV-GFP-1056 (Fig. 1e–g). These results are in agreement with previous findings^28^ and suggest that a rather homogeneous distribution and expression of the recombinant vector occurred throughout mycelia of *P*. *infestans*, demonstrating the possibility to infect oomycetes with a plant virus and express protein ectopically in their cells.

**Figure 2:**
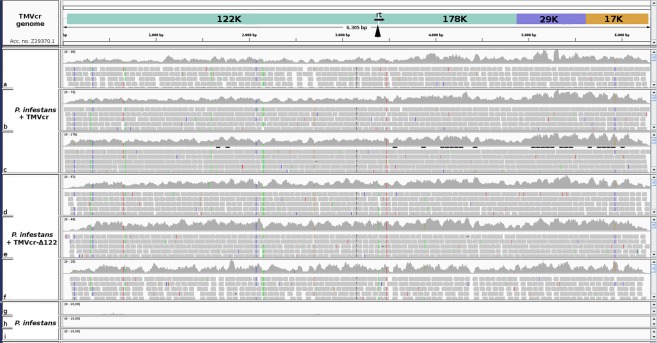
TMVcr and TMVcr-Δ122 retain their primary genome structure after replication in *P*. *infestans*. Coverage achieved after mapping sRNA reads produced by Illumina sequencing, against the TMVcr reference sequence Z29370.1. Quality filtered reads were generated from sequencing of cDNA libraries from three biological replicates of *P*. *infestans* mycelia inoculated with purified preparations of TMVcr (**a**,**b**,**c**), TMVcr-Δ122 (**d**,**e**,**f**) and from mock-inoculated WT cultures (**g**,**h**,**i**). Reads were aligned with HISAT2 2.0.5 and visualized using the Integrative Genomics Viewer (IGV) tool^48,49^. Arrowhead points 3390 nt position in which TMVcr and TMVcr-Δ122 genomes differ in the p122 amber stop codon TAG replaced with TAA.

We inoculated liquid cultures of *P*. *infestans* with purified preparations of TMVcr and TMVcr-Δ122. The presence of the two viruses in mycelia of *P*. *infestans* was confirmed by sequencing cDNA libraries generated from three biological replicates collected at 10 dpi and from mock-inoculated WT cultures. Sequences were generated by an external Illumina sequencing service (Genewiz) and 178 million raw single reads were produced from nine libraries with an average of 19.7 million reads per sample. Reads quality was assessed using FastQC^29^ and reads were trimmed with Trimmomatic, version 0.36^30^ that, after filtering for rRNA and adapters, yielded 175 million of 51 nt high quality reads. Consensus sequences of TMVcr and TMVcr-Δ122 were reconstructed on the available TMVcr genome (Z29370.1) by aligning reads by HISAT2 2.0.5 software^31^. BLASTx searches in the GenBank Virus Reference Sequence Database showed that the consensus sequences from mycelia inoculated with TMVcr and TMVcr-Δ122 had 97.5%, 98.1% and 97.5% amino acid identity with putative replicase, movement protein and coat protein, respectively, encoded by TMVcr or TMVcr-Δ122. The consensus sequence of TMVcr-Δ122 confirmed that, in mycelia of *P*. *infestans*, the amber stop codon of p122 at position 3390 retained the mutation to a tyrosine codon as in the original preparation used for viral inoculation (Fig. 2). The presence of TMVcr and TMVcr-Δ122 in *P*. *infestans* was confirmed by RT-PCR targeting TMVcr replicase, which yielded the expected amplified product of 324 bp and viral replication was confirmed by detection of TMVcr and TMVcr-Δ122 subgenomic RNA in northern blots (see Supplementary Fig. S2). Thus the feasibility and viability of the reporter system based on TMVcr and its cr-Δ122 mutant in mycelia of *P*. *infestans* was validated.

### Time-course analysis reveals differential accumulation of TMVcr and TMVcr-Δ122 RNA in mycelia of *P*. *infestans*

**Figure 3:**
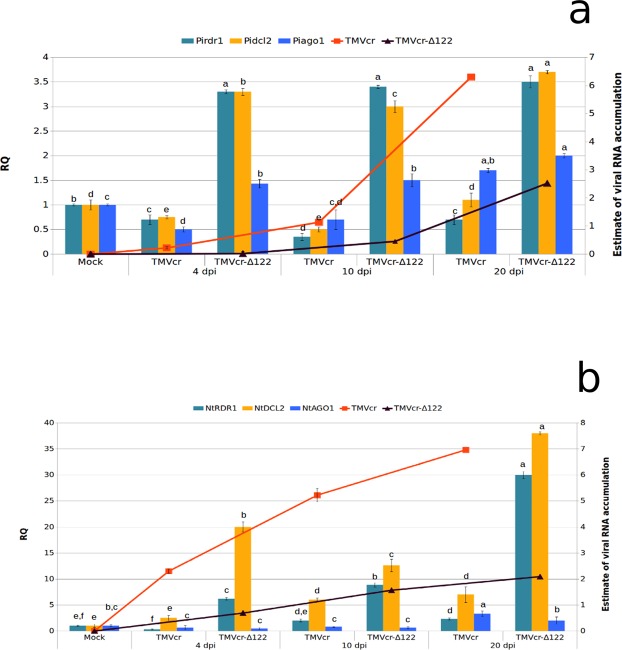
RNA of TMVcr and TMVcr-Δ122 accumulate in mycelia of *P*. *infestans* and in tobacco plants and trigger in different ways host RNAi. Load of viral RNA (lines) was estimated by qPCR and expressed as means of two independent experiments. Samples were collected from liquid cultures of *P*. *infestans* (**a**) or systemically infected leaves of *N*. *tabacum* cv Samsun (**b**) at 4, 10 and 20 dpi with TMVcr (red line) or TMVcr-Δ122 (black line). Each point in the line chart represents the average of three biological replicates for each of the two experiments and bars indicate the standard error of the mean (SEM). The figure shows also the relative quantity (RQ) of *Pirdr*1, *Pidcl*2 and *Piago*1 (**a**) and *NtRDR*1, *NtDCL*2 and *NtAGO*1 (**b**) transcripts (columns chart) in total RNA preparations obtained from mycelia from liquid cultures of *P*. *infestans* (**a**) or systemically infected leaves of *N*. *tabacum* cv Samsun (**b**) collected at 4, 10 and 20 dpi with TMVcr or TMVcr-Δ122. The values were normalized relative to the level of the *actinA* and *GAPDH* mRNAs used as housekeeping genes for *P*. *infestans* and *N*. *tabacum*, respectively. Columns represent mean RQ values from three biological replicates for each of the two experiments and different letters represent statistically significant differences values according to one-way ANOVA analysis and Tukey’s post-hoc test (*P* < 0.05). Vertical bars on columns represent SEM among replicates.

Progression in the accumulation of RNA of TMVcr and of its Δ122 mutant was estimated in total RNA preparations extracted from mycelia of *P*. *infestans* collected from liquid cultures at 4, 10 and 20 dpi and, for comparison, from systemically infected leaves of tobacco plants at the same sampling time. In *P*. *infestans*, samples collected between 4 and 20 dpi showed a 5.1-fold increase of TMVcr RNA from 4 to 10 dpi and a further 5.6-fold increase from 10 to 20 dpi reaching an overall 31.5-fold increase over the time course analysed. At each time point, the accumulation of the mutant TMVcr-Δ122 RNA was consistently lower than that of TMVcr; in particular, it was below the limit of detection at 4 dpi and 2.5-fold lower than that of TMVcr at 10 and 20 dpi (Fig. 3a). On the whole, these results suggest the progressive albeit different accumulation of both viruses in mycelia of the oomycete.

**Figure 4:**
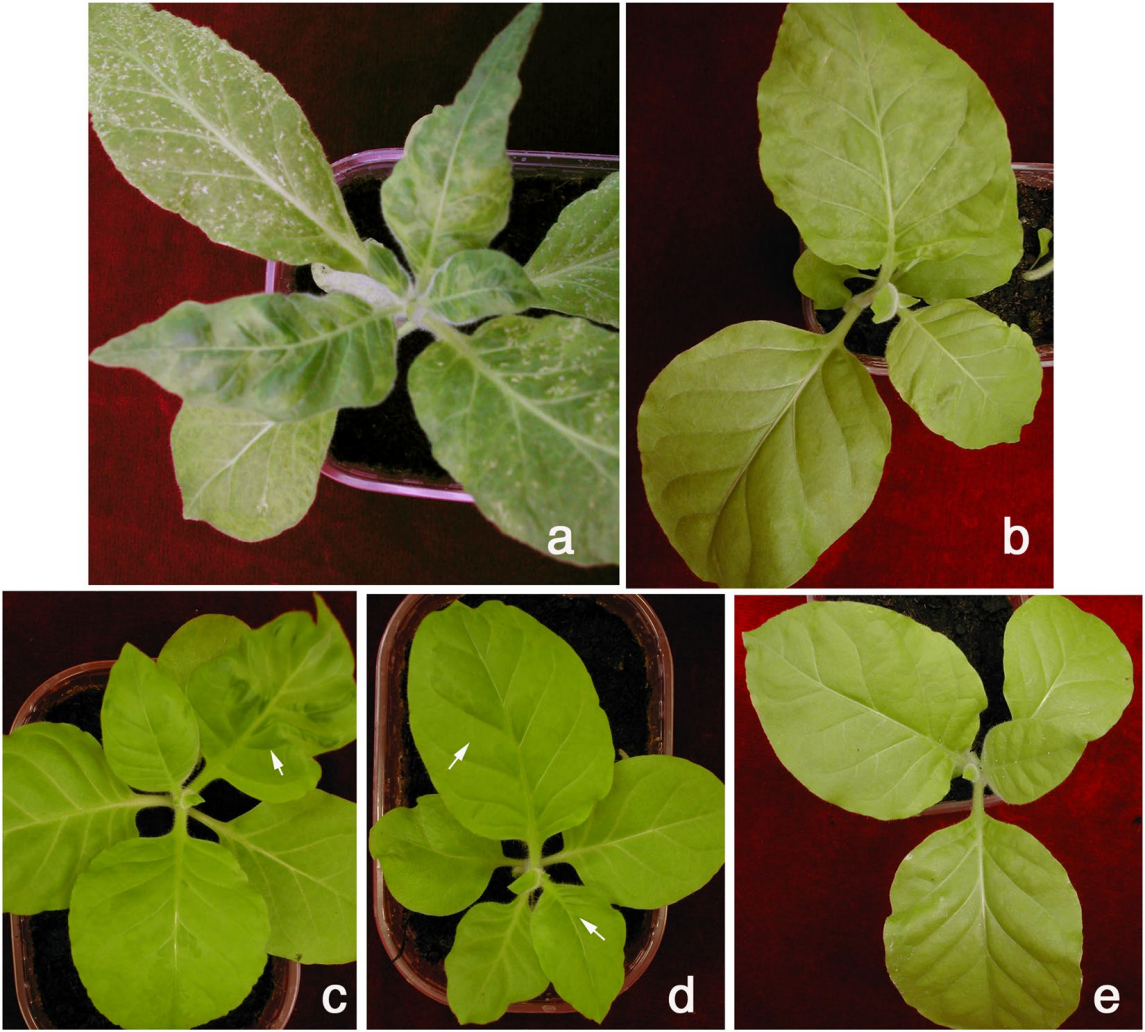
TMVcr and TMVcr-Δ122 retain infectivity in tobacco, after passaging in mycelia of *P*. *infestans*. (**a**) Mosaic and severe leaf deformation and mild mosaic (**b**) shown by tobacco plants rub-inoculated with plant sap obtained from leaves of plants infected by TMVcr and TMVcr-Δ122, respectively, compared with those induced by rub-inoculation with mycelia collected from liquid cultures of *P*. *infestans* at 10 dpi with TMVcr (**c**) and TMVcr-Δ122 (**d**). (**e**) Mock-inoculated tobacco plants.

A similar pattern was obtained from total RNA preparations extracted from leaf discs cut from systemically infected leaves of three tobacco plants, representing three biological replicates for each time point. Compared to *P*. *infestans*, in plants there was a more gradual accumulation of TMVcr RNA with 2.5-fold increment between 4 and 10 dpi and 1.4-fold increment between 10 and 20 dpi. The overall accumulation of TMVcr RNA in plants was higher than in *P*. *infestans* for each of the corresponding time points and at each time point accumulation of TMVcr RNA in plants was 3.4-fold higher than that of TMVcr-Δ122 (Fig. 3b) and congruent with enhanced disease symptom severity (Fig. 4a,b).

To test whether viruses retained infectivity in plants after replication in *P*. *infestans*, mycelia grown in liquid medium were collected at 20 dpi after treatment with either TMVcr or TMVcr-Δ122 and subcultured onto PA agar at weekly intervals for three weeks. Sporangia picked-up from the third subculture were transferred in liquid medium for a further 10-days growth after which mycelia were collected, treated with sodium hypochlorite, crushed in phosphate buffer and rub-inoculated onto Samsuntobacco plants. Infected plants developed disease symptoms of different severity between 12 and 15 dpi. Plants inoculated with mycelia infected by TMVcr developed severe leaf malformation and bubbling (Fig. 4c) whereas those inoculated with mycelia infected by TMVcr-Δ122 developed only mild mosaic (Fig. 4d). Plants rub-inoculated with a mixture obtained by crushing mycelia of *P*. *infestans* not exposed to inoculation with TMVcr or TMVcr-Δ122 remained free of symptoms (Fig. 4e).

**Figure 5:**
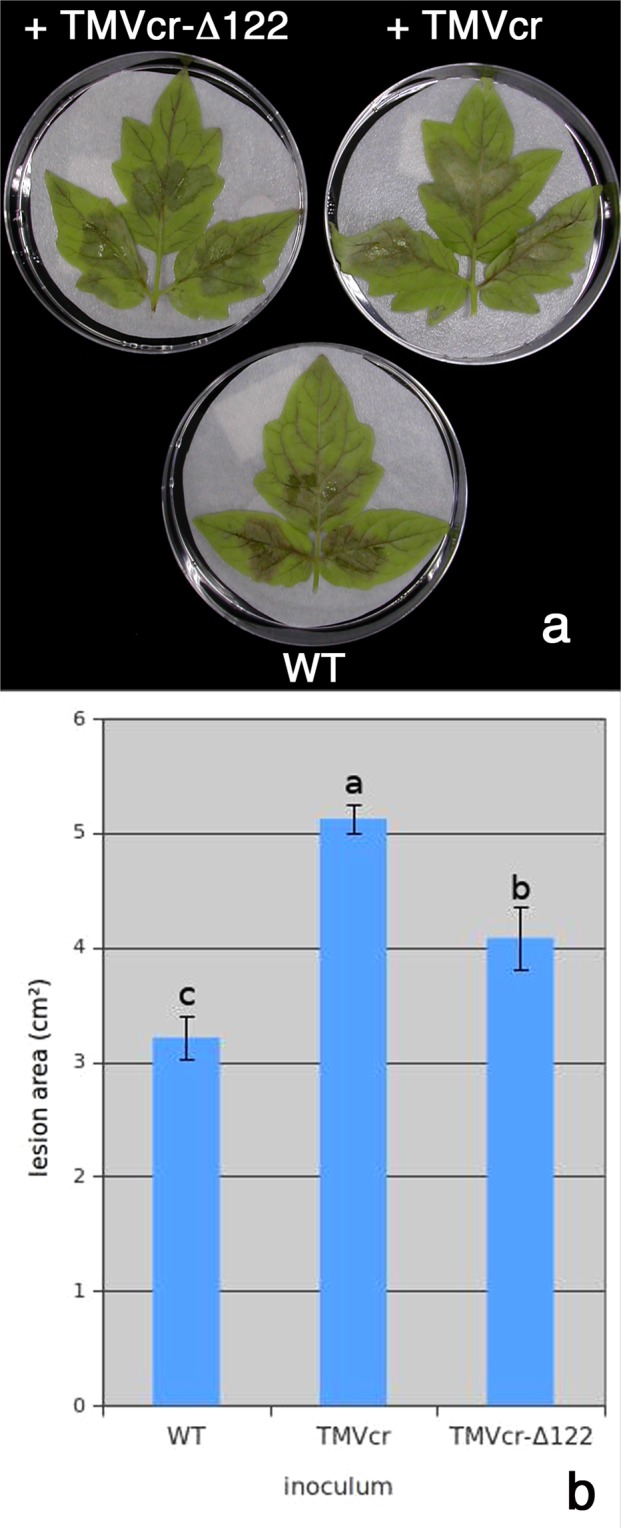
Replication of TMVcr and TMVcr-Δ122 alters the pathogenicity of *P*. *infestans*. (**a**) Lesions and mycelium proliferation on detached tomato leaflets at 7 dpi with 30 μl droplets of 1 × 10^4^ sporangia suspension collected from cultures of *P*. *infestans* established in PA plates from liquid cultures exposed to TMVcr and TMVcr-Δ122 inoculum. WT = *P*. *infestans* culture not exposed to viral inoculum. (**b**) Estimated size of the lesions induced by different inocula. Columns represent mean values of the size of a group of lesions from three biological replicates of three leaflets each from two separate experiments and letters represent statistically significant differences values, based on one-way ANOVA for *P* < 0.05 (Tukey post-hoc test). Vertical bars represent the SEM (**b**).

We next determined whether viral infection could enhance or reduce *P*. *infestans* pathogenicity in detached tomato leaflets. After inoculation, disease symptoms gradually appeared and at 7 dpi consisted of lesions of different size and gravity depending on the inoculum used (Fig. 5a). Compared with the necrotic disease symptoms that appeared on leaflets inoculated with virus-free WT *P*. *infestans*, those induced by *P*. *infestans* infected by either TMVcr or TMVcr-Δ122 did not induce necrosis, were larger in size and, at least in the case of TMVcr infection, with higher production of sporangia (Fig. 5a,b). To some extent, these results suggest that infection of TMVcr or of its mutant stimulated the growth of *P*. *infestans* but reduced its pathogenicity.

### Viral infection mediates the virus-responsive induction of RNAi hallmark genes and the synthesis of siRNAs in *P*. *infestans*

To understand how *P*. *infestans* responded to infection of TMVcr and TMVcr-Δ122 we used qPCR to estimate the relative abundance of *Pidcl2*, *Piago1* and *Pirdr*1 transcripts encoding candidate proteins in the silencing pathway of *P*. *infestans*^23^. These genes were selected because they are involved, respectively, in the antiviral defense response to dice dsRNAs, to recruit vsiRNAs for the assembly of the RISC complex and to amplify and spread the silencing signal. Additionally these genes were selected because the p122 protein of TMVcr suppresses RNAi by binding preferentially to double-stranded 21-nt vsiRNA, thus inhibiting RISC assembly. For comparison, the transcription profile of the respective counterparts *NtDCL2*, *NtAGO1* and *NtRDR1* was estimated by qPCR in samples collected at the same time points from plants of *N*. *tabacum* cv Samsun infected with TMVcr or TMVcr-Δ122. Variations in the transcription profiles were monitored in mycelia and plant leaf samples collected at 4, 10 and 20 dpi in two separate experiments. At each sampling time RNA preparations were obtained from three biological replicates and a technical replicate was included for each biological replicate. Melting curves of each reaction showed a single peak suggesting that there was no amplification of non-target fragments. Compared to mock-inoculated controls, infection of TMVcr in *P*. *infestans* did not significantly induce accumulation of transcripts of the three genes considered with the exception for a 1.7-fold upregulation of *Piago*1 at 20 dpi, which corresponded to a 3.5-fold increase in the accumulation of viral RNA (Fig. 3a). Induction and upregulation of the three genes was more gradual in tobacco and with a significant correlation between the accumulation of the viral RNA and relative abundance of the transcripts (Fig. 3b). On the contrary, high transcriptional induction was observed in both *P*. *infestans* and tobacco infected by TMVcr-Δ122 for *Pidcl*2, *Pirdr*1 (up to 3.7-fold at 20 dpi), and *NtDCL*2 (up to 38-fold) and *NtRDR*1 (up to 20-fold), respectively. Upregulation was also observed in the transcription of *Piago*1 and *NtAGO*1 but to a lesser extent (approx 2-fold for both genes at 20 dpi) (Fig. 3a,b).

**Figure 6:**
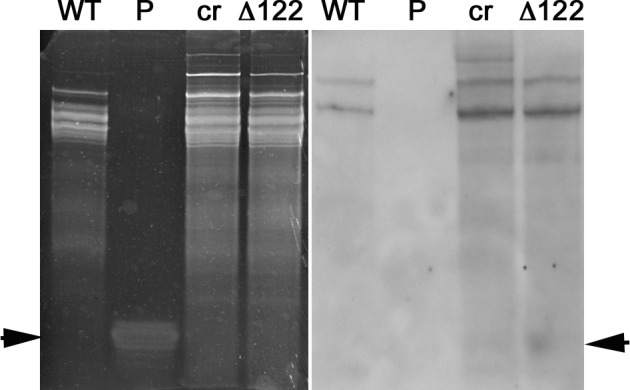
*P*. *infestans* produces virus-specific siRNAs in response to viral infection. Detection of virus-specific siRNAs in total RNA preparations enriched in sRNAs extracted from mycelia of *P*. *infestans* at 10 dpi with specific for TMVcr (cr) and TMVcr-Δ122 (Δ122). These bands were not detected in RNA preparations extracted from mycelia of *P*. *infestans* not exposed to co-incubation with viral preparations (WT). Panel (**a**) shows an image of the Gel red-stained 15% polyacrilamide gel as loading control. Panel (**b**) shows hybridization of hydrolyzed DIG-RNA probes for TMVcr replicase and CP. Arrowheads point migration of 21-nt ssDNA primers as size markers (panel **a**) and migration of siRNAs (panel **b**).

**Figure 7:**
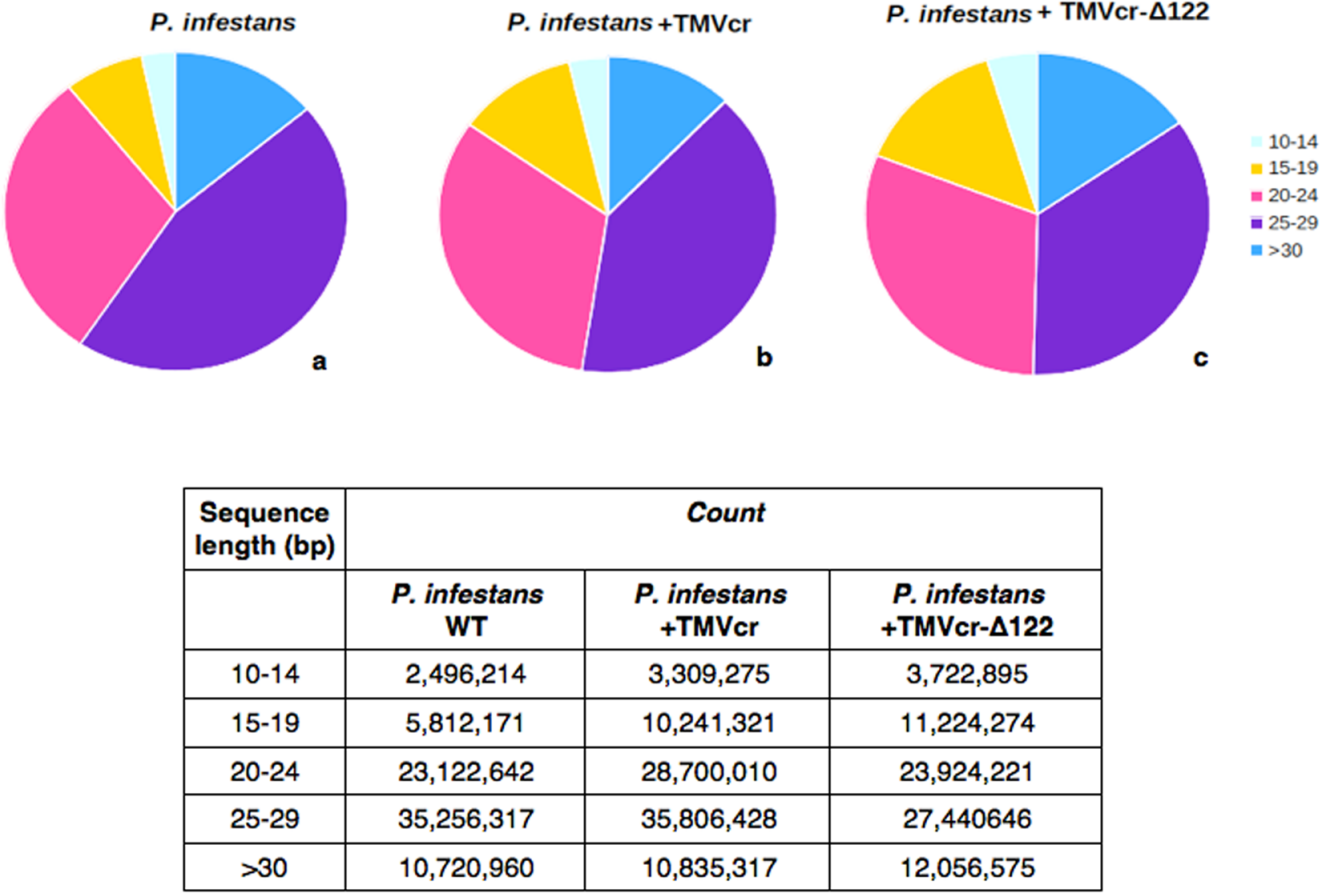
Small RNA reads in *P*. *infestans* follow a bimodal distribution. Distribution of small RNAs in *P*. *infestans* mock-inoculated (**a**) and after 10 days co-incubation with TMVcr (**b**) or TMVcr-Δ122 (**c**). Graphs are color-coded by small RNA size based on the legend. Inset shows number of reads of small RNA populations for each condition tested.

**Figure 8:**
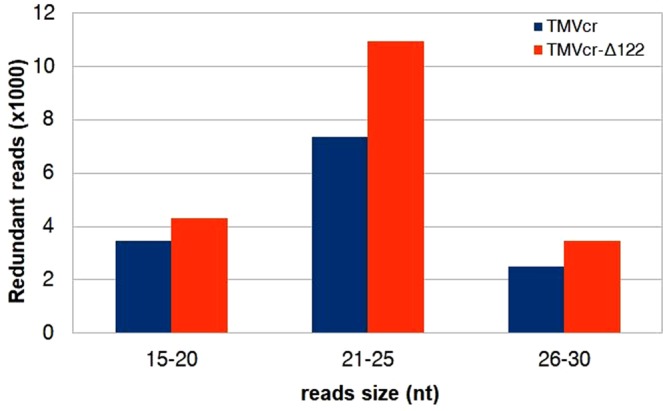
*P*. *infestans* responds to viral infection with synthesis of specific vsiRNAs. Size distribution and abundance of vsiRNAs redundant reads derived from TMVcr and TMVcr-Δ122 produced by *P*. *infestans* after 10 days co-incubation with viral inocula.

Prior studies indicated that gene silencing in *Phytophthora* spp transformants involves accumulation of siRNAs homologous to the sequence of the target gene^32,33^. We therefore tried to isolate vsiRNAs in RNA preparations extracted from mycelia of *P*. *infestans* taken at 10 dpi with TMVcr or TMVcr-Δ122. The siRNAs were identified via northern blot analysis using DIG-RNA probes for TMVcr replicase and CP. The results indicate that mycelia of *P*. *infestans* infected by TMVcr or TMVcr-Δ122 but not of the mock-inoculated WT culture contain vsiRNAs specific for TMVcr and TMVcr-Δ122 (Fig. 6). The bands had the same mobility of a 21-nt ssDNA primer used as marker. Other hybridization signals visible on the blot corresponded very likely to be rRNAs as they were also detected in mycelia of *P*. *infestans* not exposed to co-incubation with viral preparations. Thus they must be considered non-specific and probably due to the low-stringency hybridization protocol required by the hydrolysed probes. Accumulation of vsiRNAs specific for TMV-cr and TMVcr-Δ122 was also estimated by high-throughput sequencing of small RNAs extracted from mock-inoculated WT mycelia or exposed for 10 days to co-incubation with TMVcr (P.i. + TMVcr)) and its Δ122 mutant (P.i. + TMVcr-Δ122). Two biological replicate samples for each condition were sequenced yielding an average of 96 million redundant reads. After parsing from adaptors, reads that aligned without mismatches to *P*. *infestans* genome had an overall alignment rate of 86.5%, for *P*. *infestans* mock-inoculated WT, and of 85.98% and 84.46% for P.i. + TMVcr and P.i. + TMVcr-Δ122, respectively (Supplementary Table S1). The size distribution of small RNAs was bimodal in all the three conditions tested with one peak representing 20–24 nt reads and the other 25–29 nt reads, which was also the most abundant between the two size classes (Fig. 7). All these data were in good agreement with the analysis of endogenous small RNA population of *P*. *infestans*, reported by Fahlgren and coworkers^22^. Percentage of redundant vsiRNA reads among total reads parsed from adaptors matching the entire TMVcr and TMVcr-Δ122 genome (see Supplementary Fig. S3) were 0.02% (26,500 reads) and 0.03% (27,973 reads), respectively. Size distribution of the 15- to 30 nt redundant vsiRNA reads showed a predominance of 21–25 nt in TMVcr and its Δ122 mutant (Fig. 8), a figure that is in agreement with vsiRNA size estimates from northern blot analysis.

Taken together, these results suggest that TMVcr-Δ122 is more influential in the up-regulation of the RNAi genes than TMVcr, both in *P*. *infestans* and in tobacco. This up-regulation coincided with low accumulation of viral RNA in the two hosts and very mild symptom induction in tobacco and was congruent with the proposed inability of TMVcr-Δ122 to suppress RNAi. Csorba *et al*.^24^ demonstrated that the p122 protein of TMVcr suppresses RNAi by binding preferentially to double-stranded 21-nt siRNA thus inhibiting RISC assembly like the majority of the VSRs^34^. Isolation of vsiRNAs was attempted three times from mycelia of *P*. *infestans* but the method probably yielded small amounts of low-molecular-weight RNAs, which remained below the limit of detection. However, in a fourth attempt, vsiRNAs for TMVcr and TMVcr-Δ122 were detected by either northern blot or Illumina Hiseq sequencing. Results showed a 1.55-fold increase of vsiRNAs in P.i. + TMVcr-Δ122 in comparison with vsiRNAs detected in P.i. + TMVcr (Fig. 8) which seems congruent with the low accumulation of the mutant TMVcr-Δ122 RNA in mycelia of *P*. *infestans* at 10 dpi (Fig. 3a). Hence, induction of RNAi by viral infection in *P*. *infestans* was also corroborated by the detection and different accumulation of vsiRNAs. The screening method we applied provided evidence that RNAi could serve as an antiviral defense mechanism also in *P*. *infestans*. The demonstration here that, similarly to tobacco plants, the accumulation of TMVcr-Δ122 but not of TMVcr was affected by and coincident with the high induction of *Pidcl*2 provided preliminary support for the identification of *Pidcl*2 as a primary component of the antiviral pathway also in *P*. *infestans*. The upregulation of *Pirdr*1 and *NtPRDR*1 upon infection by TMVcr-Δ122 was also congruent with the inability of this virus to suppress RNAi. In *P*. *infestans Pirdr*1 is thought to be involved in the amplification of the silencing signal through the synthesis of further dsRNA primed by the residual strand of vsiRNAs not incorporated in RISC^23^. Thus, results of our study are in agreement with the upregulation of this gene if larger amounts of vsiRNAs are produced as consequence of the inability of TMVcr-Δ122 to suppress RNAi as well as with its downregulation in the case of TMVcr infection that codes for a functional TMV p122 suppressor.

This study reveals also the ability of a plant virus to infect *P*. *infestans* and documents the potential of a plant pathogen to jump to a host species belonging to a different kingdom. Oomycetes such as *P*. *infestans* are much more closely related phylogenetically to plants than they are to fungi thus it would be relatively less surprising that a plant virus would infect and trigger RNA silencing in oomycetes than in the same finding in fungi^27^. However the recently reported infection of the plant virus cucumber mosaic virus in *Rhizoctonia solani* isolated from potato plants in the field^35^, the experimental evidence that a number of taxonomically different plant viruses may infect, replicate and persist in *C*. *acutatum* and *P*. *infestans*^28^ and the ability of a mycovirus of an endophytic fungus to replicate in plant cells^36^ suggest that host-virus interaction among mycoviruses, oomycete viruses and plant viruses may operate on a similar basis. This hypothesis is also corroborated by the notion that the same taxonomic family may include viruses that infect animals, plants, fungi oomycetes and protozoa (reviewed in^37,38^). To date, most of the mechanisms for such adaptation are still unknown.

In conclusion results from this study revealed that, similarly to plants, a plant virus infection activates an RNAi pathway in *P*. *infestans* by inducing the overexpression of the dicer and rdrd genes and synthesis of vsiRNAs through an RNAi mechanism that is sensitive to the VSRs coded by the virus. Thus, the evidence reported here may expand the information about RNAi and VSRs as trans-kingdom complementary mechanisms. In addition, results from this study highlight the utility of plants viruses for functional genomic studies in filamentous fungi^39,40^. Besides of being a new piece of work, the system described here pushes forward also the understanding on mechanisms of pathogen specialization and adaptation to new host species^35^. In this framework, this study improves the knowledge base on the biological and molecular aspects of the TMV and *P*. *infestans* interactions and condenses this knowledge base into outputs delivered through the TMV-*P*. *infestans* model, which is expected to be a new and easy-to-use tool for understanding mechanisms that can be useful for the biocontrol or at least shutting down virulence genes of this important plant pathogen.

## Methods

### *Phytophthora infestans* strain and culture conditions

The *Phytophthora infestans* isolate 96.9.5.1 (mating type A1) used in this study was kindly provided by Dr. David Cooke (The James Hutton Institute, Invergowrie, Dundee, Scotland) and was maintained (16 °C) on pea agar (PA) medium^41^. To set up liquid cultures, sporangia were collected from 3-week-old cultures on 90 mm PA plates by adding sterile distilled water (7 ml) and by gently rubbing mycelium with a glass rod. The suspension was adjusted to 1 × 10^5^ sporangia/ml using a haemocytometer and zoospores released by chilling (4 °C for 2 h) followed by incubation (1 h) at room temperature. Zoospores suspension was subsequently inoculated in liquid pea medium^41^ supplemented with ampicillin (50 mg/ml) and rifampicin (30 mg/ml) and grown under continuous shaking (19 °C, 100 rpm).

### Viruses and inocula

Full-length clones of the crucifer-infecting strain of tobacco mosaic virus (TMVcr) and of its substitution mutant TMVcr-Δ122 and a culture of *Agrobacterium tumefaciens* C58C1/pBIN61 harboring the plasmid pBIN-p122 were kindly provided by Dr. József Burgyán (National Agricultural Research and Innovation Center, Agricultural Biotechnology Center Gödöllő, Hungary). The TMVcr-Δ122 had the amber stop codon TAG of the 122-kDa replicase subunit (p122) sequence replaced with the TAA codon for tyrosine to inhibit the siRNA binding activity of p122^24^. Inocula of TMVcr and TMVcr-Δ122 were prepared in *Nicotiana occidentalis* by rub-inoculation of a biologically active transcript synthesized from the *Pml*I-linearized plasmids pUC-TMVcr and pUC-TMVcr-Δ122^23^ with T7 RNA polymerase and the mMessage mMachine kit (Ambion), following the protocol of the manufacturer. Two true leaves of four plants of *N*. *occidentalis* were inoculated each with the transcription (30 μl) mixture obtained from pUC-TMVcr whereas to activate replication of the transcript of TMVcr-Δ122 we followed the protocol of Csorba *et al*.^24^. Briefly, leaves of *N*. *occidentalis* were first agroinfiltrated with a preparation of *A*. *tumefaciens* C58C1/pBIN-p122 to express a functional p122 protein and after two days the same leaves were inoculated with the transcription mixture (30 μl) obtained from pUC-TMVcr-Δ122. Plants agroinfiltrated with a preparation of *A*. *tumefaciens* carrying a pBIN empty plasmid and after two days with the transcription mixture (30 μl) obtained from pUC-TMVcr-Δ122, served as control. All the plants were grown in a greenhouse (22 ± 2 °C, 16-h photoperiod) and monitored daily for symptom appearance. Plant sap extracted in phosphate (Na_2_-K) buffer (100 mM, pH 7.2) from systemically infected leaves collected at 10 days post inoculation (dpi) was used to inoculate plants of *N*. *tabacum* cv Samsun for virus maintenance and purification. Viral particles of TMVcr and its mutant TMVcr-Δ122 were purified from tobacco leaves as described by Lot *et al*.^42^ and stored at −20 °C in 50 mM NaCl, 30% glycerol solution until used. Both the strands of an amplified product from the viral replicase (see below) were sequenced by an external sequencing service to assess conservation of the TAA mutation after multiplication in plants and purification. Prior to inoculation in liquid cultures of *P*. *infestans*, infectivity of purified virus preparations of TMVcr and its mutant TMVcr-Δ122 was evaluated by rub-inoculation onto *N*. *tabacum* cv Samsun.

To inoculate *P*. *infestans*, purified preparations of TMVcr or TMVcr-Δ122 (50 μl, 1 μg/ml) were added to sporangia growing in pea broth medium^41^ (100 ml) supplemented with ampicillin (50 mg/ml) and rifampicin (30 mg/ml). Cultures mock-inoculated with 50 μl of NaCl-glycerol solution (50 mM NaCl, 30% glycerol) served as wild-type (WT) negative control. All cultures were prepared in triplicates and incubated under continuous shaking (19 °C, 100 rpm).

### Nucleic acid extraction and analysis

Mycelia of *P*. *infestans* co-incubated with purified virus preparations or systemically infected leaves of *N*. *tabacum* were collected at 4,10 and 20 dpi with TMVcr or TMVcr-Δ122. To remove virus particles adhering externally to hyphae, mycelia were harvested by filtering through sterilized Whatman No.1 filter paper, treated (30 s) with a commercial solution of sodium hypochlorite (2% vol/vol) and washed extensively with sterile distilled water^26^. To assess the efficacy of sodium hypochlorite treatment in removing virus particles adhering externally to mycelia of *P*. *infestans*, wash samples were rub-inoculated onto *N*. *occidentalis* and Samsun tobacco plants after dialysis (10 mM Tris-HCl, 1 mM EDTA, pH 8) to eliminate residues of sodium hypochlorite.

Total RNA was extracted with Eurogold RNA pure (Euroclone) from freeze-dried mycelia or tobacco leaves (100 mg) ground to a fine powder with liquid nitrogen, following the protocol of the manufacturer. RNA was suspended in RNase-free water and the concentration was adjusted to 1 μg/μl. RNA preparations were separated by electrophoresis (1.2% agarose gel in 90 mM Tris, 90 mM boric acid, 1 mM EDTA) and stained with Gel-red (Biotium). When necessary, northern blots were prepared and subjected to hybridization with digoxigenin (DIG)-labeled DNA probes specific for the sequence coding for the coat protein (CP) of TMVcr (see below) following the protocol of Minutillo *et al*.^43^. The ChemiDoc system apparatus and Quantity One software (Bio-Rad Laboratories) were used to detect the chemiluminescent signals.

Standard reverse-transcription polymerase chain reactions (RT-PCR) were used throughout to detect the TMVcr and TMVcr-Δ122 in mycelia of *P*. *infestans* or plants. Reactions were carried out with primer pairs mapping to TMVcr sequences (Acc. no. Z29370.1) coding for coat protein (For 5′-CAGTGCATATCGGCATTGTC-3′, Rev 5′-CGTGACTCCTCTTCCGTCTC-3′) or TMVcr replicase (For 5′-AAGGTGGTGGTGGTTTCGACAAG-3′, Rev 5′ACTTGTGGTTGGCATCTTCC-3′). PCR conditions were: denaturation (4 min, 94 °C) followed by 35 cycles of denaturation (30 s, 94 °C), annealing (30 s, 50 °C) for TMV-cr coat protein and 55 °C for TMV-cr replicase and synthesis (30 s, 72 °C). Final elongation step was for 5 min at 72 °C. Purity and size of the amplified products was estimated by electrophoresis (1.2% agarose gel, TBE buffer, Gel-red staining). For probe synthesis, amplified products (324 bp) from TMVcr replicase (REP) and from TMVcr coat protein (CP) (227 bp) were ligated to p-GEM-T- Easy vector (Promega) and cloned in DH5α cells to yield pREPcr and pCPcr plasmids, respectively.

For detection of vsiRNAs, these were extracted using either Eurogold RNA pure (Euroclone) or the mirVana miRNA kit (Ambion) from freeze-dried mycelia (100 mg) collected at 10 dpi and ground to a fine powder with liquid nitrogen. RNA was separated by electrophoresis through denaturing gels (15% polyacrylamide, 7 M urea), and electroblotted for 1 h at 15 V in 0.5TBE to positively-charged nylon membranes (Roche). Membranes were probed with UTP-DIG–labelled probes for TMVcr REP and CP transcribed from pREPcr and pCPcr plasmids, respectively, and hydrolyzed as described^27^. Hybridizations and washes were conducted at 42 °C and chemiluminescent signal acquired by ChemiDoc (Bio-Rad Laboratories). Two synthetic DNA oligonucleotides of 21-, and 25-nt long were used as molecular size markers.

The same small RNA preparations were also sent to an external sequencing service (Genewiz) to prepare libraries for sequencing on the Illumina Hiseq platform. Sequencing reads produced from six libraries with an average of 96 million reads per sample, were quality assessed with FastQC^29^, parsed to remove 5′TGGAATTCTCGG3′ adaptors with Trim Galore!(Galaxy Version 0.4.3.1) and aligned to *P*. *infestans* genome using BOWTIE2 (Galaxy Version 2.3.4.2). The annotated genome for *P*. *infestans* was downloaded from the Broad Institute website^44^.

### Quantitative real-time PCR

Quantitative real-time PCR (qPCR) was conducted using the Applied Biosystems StepOne™ Real-Time PCR System and Fast SYBR®Green Master Mix (Applied Biosystems). Absolute qPCR was performed to monitor TMVcr CP accumulation at 4, 10 and 20 dpi in *P*. *infestans* and *N*. *tabacum*. Samples were run in triplicate and reactions were set up in a total volume of 10 μl using 66 ng of cDNA (66 ng) and primers (250 nM each) described above. Three replicates of serially diluted preparations (5-fold) of the pREPcr plasmid were used to construct standard curves. The cycling conditions were: 95 °C for 20 sec; 40 cycles at 95 °C for 3 sec and 60 °C for 30 sec. Specificity of the qPCR products was confirmed by analysis of the dissociation curves. Results were evaluated using the StepOne™ Software v2.3.

Transcript level of candidate genes encoding components of the silencing pathway were quantified in leaves of *N*. *tabacum* and in mycelia of *P*. *infestans* at 4, 10 and 20 dpi with TMVcr or TMVcr-Δ122 using the ΔΔCT method^45^ and primer pairs described by Vetukuri *et al*.^23^. Relative expression of *Dicer-like 2* (*NtDCL2*) (Acc. n. KF006308.1), *Argonaute 1* (*NtAGO1*) (Acc. n. AB542739.1) and *RNA-dependent RNA polymerase 1* (*NtRDR1*) (Acc. n. AJ011576) in *N*. *tabacum* was estimated using g*lyceraldehyde phosphate dehydrogenase* (GAPDH) (Acc. n. AJ133422) as a housekeeping gene^46^. Expression levels of *Dicer-like 1* (*Pidcl1*) (Acc. n. EEY55353), *Argonaute 1-2* (*Piago1/2*) (Acc. n. EEY67432) and *RNA-directed RNA polymerase 1* (*Pirdr1*) (Acc. n. EEY56917) in *P*. *infestans* were estimated using *actinA* (*PiactA*) as housekeeping gene^23^. Relative abundance of the transcripts was compared with mock-inoculated samples collected at the same time points from the two hosts.

### Optical and confocal microscopy

TMV replication and expression in *P*. *infestans* were confirmed by observing the green fluorescent protein (GFP) emitted fluorescence in mycelia and sporangia of *P*. *infestans* inoculated with the recombinant vector TMV-GFP-1056. The vector was grown in *N*. *occidentalis* inoculated with biologically active RNA transcripts synthesized *in vitro* from the plasmid TMV-GFP-1056^47^ obtained from Dr. Peter Palukaitis (Department of Horticultural Sciences, Seoul Women’s University, Seoul, South Korea). The TMV-GFP-1056 plasmid was linearized at the *Kpn*I site and the recombinant vector transcribed using T7 RNA polymerase and the mMessage mMachine kit (Ambion), following the protocol of the manufacturer. Observations were made using AxioPlan epifluorescent microscope (Zeiss) equipped with a mercury vapor lamp light source HBO 50 and filter combination BP450-490/FT510/LP520. Fluorescent images were acquired also from an inverted Leica LSM TSC SP2 AOBS confocal microscope (www.leica-microsystems.com) equipped with a 63X oil-immersion objective. Bright field images were acquired from the same areas.

### Pathogenicity test

For virus infectivity test in plants, mycelia of *P*. *infestans* were collected from liquid cultures at 10 dpi with TMVcr or TMVcr-Δ122, treated with sodium hypochlorite and crushed in Na_2_-K phosphate buffer (100 mM, pH 7.2). The slurry was rub-inoculated on celite-dusted leaves of Samsun tobacco. Plants inoculated with slurry obtained from mycelia of mock-inoculated wild type (WT) cultures of *P*. *infestans* collected at 10 dpi served as control. All plants were maintained in greenhouse (22 ± 3 °C, 16 h light/8 h dark photoperiod).

For the *P*. *infestans* pathogenicity test, mock-inoculated WT mycelia or mycelia inoculated with TMVcr or TMVcr-Δ122 were grown on PA plates. Sporangia were collected by washing 2-week-old cultures with distilled water and used as inoculum on UC82 tomato leaflets excised from the plants. Leaflets were surface-sterilized with sodium hypochlorite solution (2% vol/vol), washed, dried and transferred on moist filter paper in Petri dishes. Each leaflet was inoculated on abaxial side with droplets (30 μl) of sporangia suspension (1 × 10^4^) near the main vein and kept in growth chambers (16 °C, 16 h light/8 h dark photoperiod). Three leaflets placed in the same Petri dish were used as a replicate, and three plates were used for each inoculum. Disease severity was estimated by measuring the area of the developed lesion from two separate experiments.

## Supplementary information

Supplementary materials

## Data Availability

All data generated or analysed during this study are included in this published article (and its Supplementary Information files).
