# Supplementary material for: Tobacco mosaic virus infection triggers an RNAi-based response in Phytophthora infestans
Source: Sci Rep. 2019 Feb 25;9:2657. doi: 10.1038/s41598-019-39162-w (PMC6390105; doi:10.1038/s41598-019-39162-w)
Supplement: Supplementary file 1 — Supplementary materials [file 41598_2019_39162_MOESM1_ESM.pdf]

Tobacco mosaic virus infection triggers an RNAi-based response in *Phytophthora infestans.*

Tiziana Mascia[1,2 *], Rossella Labarile[1], Fiona Doohan[3], Donato Gallitelli[1,2*]

[1] Dipartimento di Scienze del Suolo della Pianta e degli Alimenti, Università degli Studi di Bari Aldo Moro, Bari, Italy

[2] Istituto del CNR per la Protezione Sostenibile delle Piante, UOS di Bari, Bari, Italy

[3]School of Biology & Environmental Science, University College, Dublin, Belfield Dublin 4, Ireland

[*] Correspondence and requests for materials should be addressed to T.M (email:tiziana.mascia@uniba.it) or D.G. (e-mail: donato.gallitelli@uniba.it)

Supplementary materials

Supplementary Table

Table S1. Distribution of total reads in DNA libraries prepared from total RNA extracted from mycelia of mock-inoculated *P. infestans* (Pi WT) or exposed to co-incubation with TMVcr (Pi+TMVcr) or TMVcr- Δ122 (Pi+TMVcr- Δ122). Figures in brackets represent percent alignment rate.

Figure S1. TMV-GFP-1056 is viable in *N. tabacum.* a) Local green fluorescence emitted by *N. tabacum* cv Samsun 4 dpi with TMV-GFP-1056. b) Mock-inoculated tobacco plant.

Figure S2. TMVcr and TMVcr-Δ122 enter and replicate in mycelia of *P. infestans.* Detection of viral RNA by northern blot hybridization with a DIG-labeled DNA probe for the TMVcr CP in 10 μg total RNA preparations extracted from mycelia collected from liquid cultures of *P. infestans* at 10 dpi with TMVcr and TMVcr-Δ122. Panel **a** Gel red- stained 1.2% agarose gel and **b** after hybridization with DIG-Labeled DNA and chemiluminescent detection. P Δ122 and P cr = RNA extracted from purified preparations of TMVcr-Δ122 and TMVcr, respectively. Pi WT = total RNA preparation extracted from mycelia of *P. infestans* not exposed to co-incubation with viral inocula.

Pi + cr and Pi + Δ122 = total RNA preparations extracted from mycelia of *P. infestans* exposed to co-incubation with TMVcr and TMVcr-Δ122, respectively. Arrows indicate the positions of the 6312 nt TMVcr genomic RNA (gRNA) and of the 1300 nt MP + CP subgenomic RNA (sgRNA). The subgenomic RNA coding for MP + CP is present in *P. infestans* extracts but not in purified preparations of (P Δ122 or P cr).

Figure S3. VsiRNAs generated in *P. infestans* after 10 dpi co-incubation with TMVcr or TMVcr-Δ122 cover the entire viral genome. Coverage achieved after mapping small RNA reads produced by Illumina Hiseq sequencing, against the TMVcr reference sequence Z29370.1. Quality filtered reads were generated from sequencing of cDNA libraries from two biological replicates of *P. infestans* mycelia inoculated with purified preparations of TMVcr, TMVcr-Δ122 and from mock-inoculated WT cultures. Reads were aligned with HISAT2 2.0.5 and visualized using the Integrative Genomics Viewer (IGV) tool[48,49]. Arrowhead points 3390 nt position in which TMVcr and TMVcr-Δ122 genomes differ in the p122 amber stop codon TAG replaced with TAA.

Table S1. Distribution of total reads in DNA libraries prepared from total RNA extracted from mycelia of mock-inoculated *P. infestans* (Pi WT) or exposed to co-incubation with TMVcr (Pi+TMVcr) or TMVcr- Δ122 (Pi+TMVcr- Δ122). Figures in brackets represent percent alignment rate.

**Reads**	**Pi WT**	**Pi+ TMVcr**	**Pi+ TMVcr-Δ122**
Total	90,726,741	105,181,332	94,208,293
Pursed from adaptors	89,752,162	103,390,390	92,787,198
Aligned exactly 1 time	12,257,988 (13.66%)	13,546,555 (13.10%)	11,342,035 (12.22%)
Aligned more than 1 time	65,150,316 (72.59%)	75,345,796 (72.88%)	67,026,576 (72.24%)
Overall alignment rate	77,408,304 (86.25%)	88,892,351 (85.98%)	78,368,611 (84.46%)

Figure S1

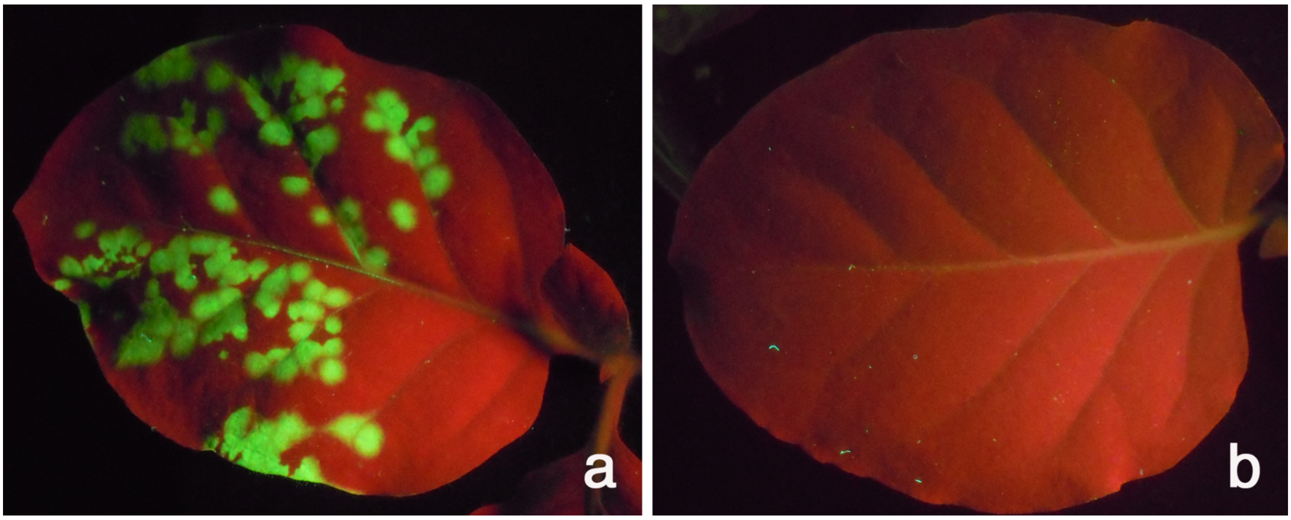

Figure S2

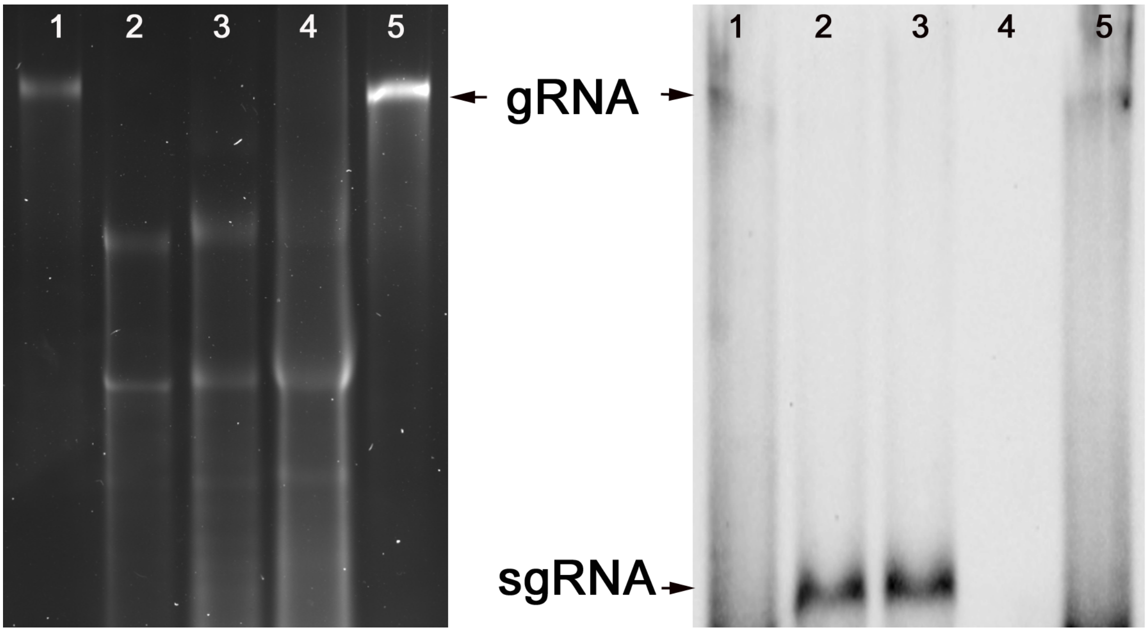

Figure S3

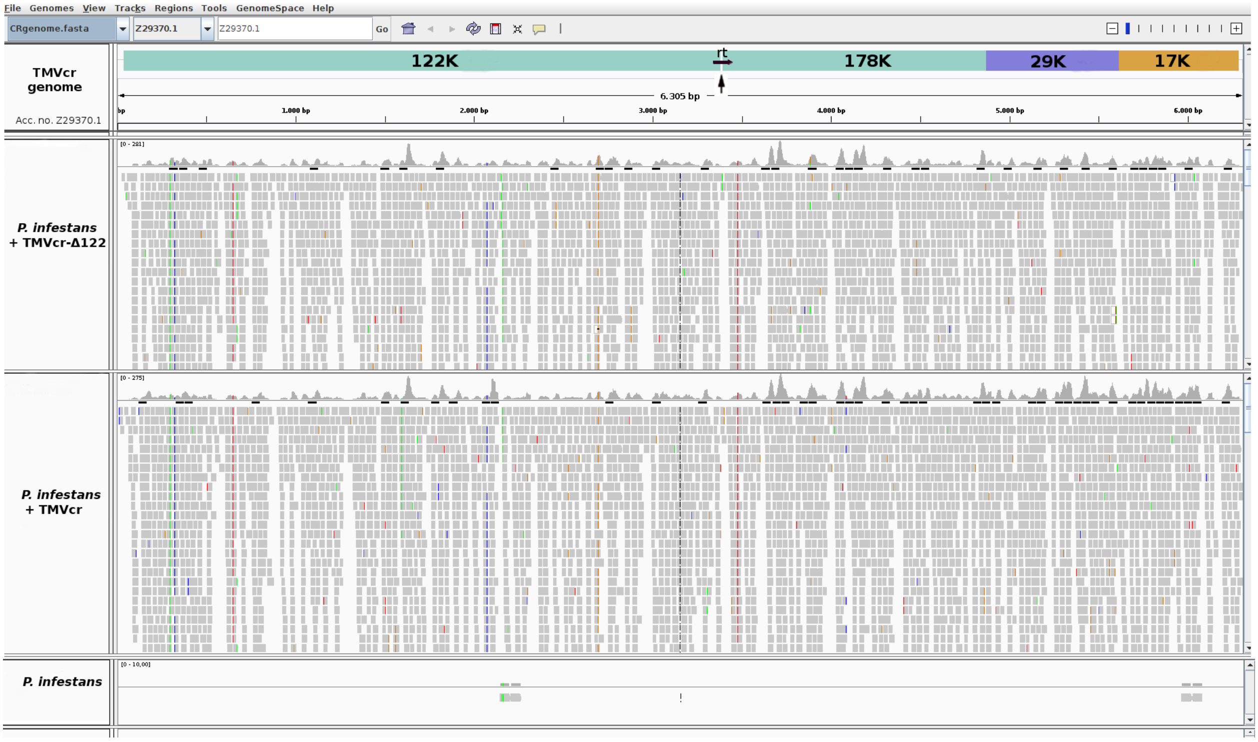
File Genomes View Tracks Regions Tools GenomeSpace Help
CRgenome.fasta
Z29370.1
Go
TMVcr genome
Acc. no. Z29370.1
122K
rt
178K
29K
17K
6.305 bp
1.000 bp
2.000 bp
3.000 bp
4.000 bp
5.000 bp
6.000 bp
P. infestans + TMVcr-Δ122
P. infestans + TMVcr
P. infestans
